# Metagenomic analyses of the gut microbiota associated with colorectal adenoma

**DOI:** 10.1371/journal.pone.0212406

**Published:** 2019-02-22

**Authors:** Keisuke Saito, Shigeo Koido, Toshitaka Odamaki, Mikio Kajihara, Kumiko Kato, Sankichi Horiuchi, Sei Adachi, Hiroshi Arakawa, Sayumi Yoshida, Takafumi Akasu, Zensho Ito, Kan Uchiyama, Masayuki Saruta, Jin-zhong Xiao, Nobuhiro Sato, Toshifumi Ohkusa

**Affiliations:** 1 Division of Gastroenterology and Hepatology, The Jikei University School of Medicine, Kashiwa Hospital, Chiba, Japan; 2 Gut Microbiota Department, Next Generation Science Institute, Morinaga Milk Industry Co., Ltd., Kanagawa, Japan; 3 Department of Endoscopy, The Jikei University School of Medicine, Kashiwa Hospital, Chiba, Japan; 4 Division of Gastroenterology and Hepatology, The Jikei University School of Medicine, Tokyo, Japan; 5 Department of Microbiota Research, Juntendo University Graduate School of Medicine, Tokyo, Japan; University of South Alabama Mitchell Cancer Institute, UNITED STATES

## Abstract

Recent studies have suggested an association between certain members of the *Fusobacterium* genus, especially *F*. *nucleatum*, and the progression of advanced colorectal carcinoma (CRC). We assessed such an association of the gut microbiota in Japanese patients with colorectal adenoma (CRA) or intramucosal CRC using colonoscopy aspirates. We analyzed samples from 81 Japanese patients, including 47 CRA and 24 intramucosal CRC patients, and 10 healthy subjects. Metagenomic analysis of the V3-V4 region of the 16S ribosomal RNA gene was performed. The linear discriminant analysis (LDA) effect size (LEfSe) method was used to examine microbial dysbiosis, revealing significant differences in bacterial abundances between the healthy controls and CRA or intramucosal CRC patients. In particular, *F*. *varium* was statistically more abundant in patients with CRA and intramucosal CRC than in healthy subjects. Here, we present the metagenomic profile of CRA and intramucosal CRC and demonstrate that *F*. *varium* is at least partially involved in the pathogenesis of CRA and intramucosal CRC.

## Introduction

Increasing evidence suggests that the human gut microbiota contributes to chronic inflammation and plays important roles in the early stage of carcinogenesis of colorectal adenocarcinoma (CRC) and colorectal adenoma (CRA) through interference with various intestinal functions [1, 2]. Chronic inflammation induced by the microbiota is associated with altered interactions between the host and the microbiota, microbial imbalance (dysbiosis), and infections with specific pathogens in patients with CRC or CRA [3]. Studies recently suggested that *Fusobacterium* spp. overall (Pan-*fusobacterium*), especially *Fusobacterium nucleatum* (*F*. *nucleatum*), are abundant in advanced CRC tissue and may contribute to invasion and metastatic proliferation [4–6]. Indeed, *F*. *nucleatum* in advanced CRC is associated with CpG island methylator phenotype (CIMP) status, microsatellite instability (MSI), and mutations in the *BRAF*, *KRAS*, *TP53*, *CHD7* and *CHD8* genes [4–7]. This evidence suggests an association of the gut microbiota with the early stage of carcinogenesis of CRC following microbial dysbiosis. Moreover, the procarcinogenic activities of specific pathogens and certain microbiota-derived metabolites [8] act on colonic epithelial cells during CRC genesis. Further research may be necessary to better understand the mechanisms that underlie the association between gut microorganisms and the early stage of carcinogenesis of CRC and CRA.

In addition to *F*. *nucleatum*, *F*. *varium* [9] has also recently gained notoriety as a gastrointestinal pathogen [10, 11]. Indeed, links between the enrichment of *Fusobacterium* spp. and the development of advanced CRC have been described [4, 12–14]. These reports suggest an association between *F*. *varium* or *F*. *nucleatum* infections and the genesis of CRC. However, little is known about microbiome profiles during the transition from normal colonic mucosae and CRA to intramucosal CRC. In this study, we focused on microbiome profiles in CRA and intramucosal CRC patients compared to those in healthy subjects. We present a metagenomic profiling study of the microbiomes of CRA and intramucosal CRC patients using colonoscopy aspirates, which can serve as a substitute for the gut microbiota in tissues [15].

## Materials and methods

### Study design and enrolled subjects

The study was reviewed and approved by the ethics committee of the Jikei Institutional Ethical Board, Jikei University School of Medicine, and the clinical study committee of Jikei University Kashiwa Hospital [No. 23–277 (6738)] on February 6, 2012. Written informed consent was obtained from each patient included in the study. All procedures were performed in accordance with the Helsinki Declaration. Eighty-one consecutive Japanese people with no previous personal history of cancer were prospectively enrolled in this study and underwent regular colonoscopy at Jikei Kashiwa Hospital. After a complete colonoscopic examination, these subjects were newly classified as healthy (n = 10), CRA patients (n = 47), or intramucosal CRC patients (n = 24). Intramucosal CRC is in its earliest stage (stage 0) and is also known as carcinoma in situ or intramucosal carcinoma. Intramucosal CRC has not yet grown beyond the inner mucosal layer of the colorectum [16, 17]. Among the 81 enrolled subjects, there were no significant differences in characteristics (sex, age, body mass index, diabetes, hypercholesterolemia, hypertension, antibiotic treatment, past history, and reason for colonoscopy) between the groups (healthy, CRA, and intramucosal CRC) (Table 1).

**Table 1:** Characteristics of enrolled patients with colorectal adenoma, intramucosal colorectal carcinoma, and healthy subjects.

Characteristics of enrolled subjects	Colorectal adenoma	Intramucosal colorectal carcinoma	Healthy subjects	*P* value
Number	n = 47	n = 24	n = 10	
Males/Females	31/16	17/7	3/7	0.260
Age (mean ± SD)	67±9	66±8	58±15	0.347
Body Mass Index (BMI) (mean ± SD)	23±3	22±3	22±3	0.159
Diabetes: n (%)	6(12.8%)	7(29.2%)	0(0)	0.257
Hypercholesterolemia: n (%)	12(25.5%)	3(12.5%)	2(20%)	0.142
Hypertension: n (%)	17(36.2%)	12(50%)	0(0)	0.308
Antibiotic treatment, any, n (%)	2(4.3%)	1(4.2%)	1(10%)	0.088
Reason for colonoscopy: n (%)				
Screening	3(6.4%)	0(0)	1(10%)	0.157
Symptoms	1(2.1%)	3(12.5%)	6(60%)	0.561
Positive for an acronym for fecal occult blood test (FOBT)	11(23.4%)	4(16.7%)	3(30%)	0.100
Control for polyps	20(42.5%)	10(41.7%)	0(0)	0.288
Polypectomy	12(25.5%)	7(29.2%)	0(0)	0.211

### Colonoscopy aspirates and DNA extraction

Colonoscopy aspirates, including the intestinal content microbiota obtained from the rectum, were collected [15]. We employed a PAXgene system (Becton Dickinson, Franklin Lakes, USA) to fix the colonoscopic aspirates. Samples obtained from 10 mL of colonoscopy aspirates were centrifuged, frozen, and stored at -80°C until analysis. Total DNA was extracted from the frozen samples as described previously [18].

### 16S microbiota analysis

The V3-V4 region of the 16S rRNA gene was first PCR-amplified using the 16S amplicon PCR forward primer 5'-CGCTCTTCCGATCTCTGTACGGRAGGCAGCAG -3' and reverse primer 5'-CGCTCTTCCGATCTGACGGACTACHVGGGTWTCTAAT -3'. A 1-μl sample of the PCR product was amplified using the following barcoded primers adapted for Illumina MiSeq: Fwd 5'-AATGATACGGCGACCACCGAGATCTACAC XXXXXXXX ACACTCTTTCCCTACAC GACGCTCTTCCGATCTCTG-3' and Rev 5'-CAAGCAGAAGACGGCATACGAGAT XXXXXXXX GTGACTGGAGTTCAGACGTGTGCTCTTCCGATCTGAC-3', where X represents a barcode base. The PCR was prepared and run, and the 16S V3-V4 amplicons were purified. The amplicon libraries were pooled in equimolar concentrations, and the V3-V4 region of the 16S rRNA gene was sequenced using a MiSeq Reagent Kit on the Illumina MiSeq platform (Shallowater, US) for paired-end sequencing as described previously [19]. The obtained sequences were analyzed using Quantitative Insights Into Microbial Ecology (QIIME) version 1.8.0, which is software that performs microbial community analysis and taxonomic classification of microbial genomes [20, 21]. Potential chimeric sequences were removed using UCHIME, and the remaining sequences were assigned to operational taxonomic units (OTUs) using ppen-reference OTU picking with a 97% threshold of pairwise identity and then classified taxonomically using the Greengenes reference database (http://greengenes.secondgenome.com/downloads/database/13_5) [22]. The microbial diversities, Shannon diversity index, and weighted and unweighted UniFrac distances were estimated using QIIME version 1.8.0 software.

### Fusobacterium-targeted analysis

A primer set specific for sequencing the DNA of *Fusobacterium* spp. by MiSeq was designed based on the internal transcribed spacer (ITS) region between the 16S rRNA and 23S rRNA genes (S1 Fig). The forward primer was 5'-CGCTCTTCCGATCTCTGGGWACCRMGTGAACTGAAACATC- 3', and the reverse primer was 5' -CGCTCTTCCGATCTGACCCTTAYGAGATWTGGTCCTC- 3'. Each 1-μl sample of DNA was amplified in triplicate using the following protocol: preheating at 94°C for 3 min, 20 cycles of denaturation at 94°C for 30 s, annealing at 50°C for 30 s and extension at 72°C for 30 s, with a final terminal extension at 72°C for 10 min. The steps from the second amplification to the assignment and to OTU picking were the same as mentioned above, and sequences were then classified taxonomically using BLASTN against the NCBI nonredundant database.

### Statistical analysis

Differences in the Shannon diversity index between the 2 groups were analyzed by t-test. Intergroup differences at the phylum, class, order, family, genus and species level in each cluster were analyzed by the linear discriminant analysis (LDA) effect size (LEfSe) method [23] with default settings on the website https://huttenhower.sph.harvard.edu/galaxy/root. LEfSe uses the two-tailed nonparametric Kruskal-Wallis test to evaluate the significance of differences in OTUs in 2 groups. A set of pairwise tests among 2 groups was performed using the unpaired Wilcoxon test. Finally, LDA was performed to estimate the effect size of each differentially abundant OTU [23, 24]. The results are expressed as the mean ± SEM. A strength of the LEfSe method compared with standard statistical approaches is that in addition to providing p values, it estimates the magnitude of the association between each OTU and the grouping categories, such as CRA, intramucosal CRC, and healthy subjects [23, 24]. For stringency, the gut microbiotas were considered significantly different if their differences had a p value < 0.05 and an LDA score (log10) > 3, i.e., one order of magnitude greater than the default of the LEfSe method [23, 24]. The Kruskal-Wallis and Cramer V tests were used to determine associations between the groups of subjects and characteristics. StatView software, version J 5.1 (SAS Institute Inc., Cary, N.C., USA), was used for all analyses.

## Results

### Overview of gut microbiota composition in patients and healthy subjects

**Fig 1:**
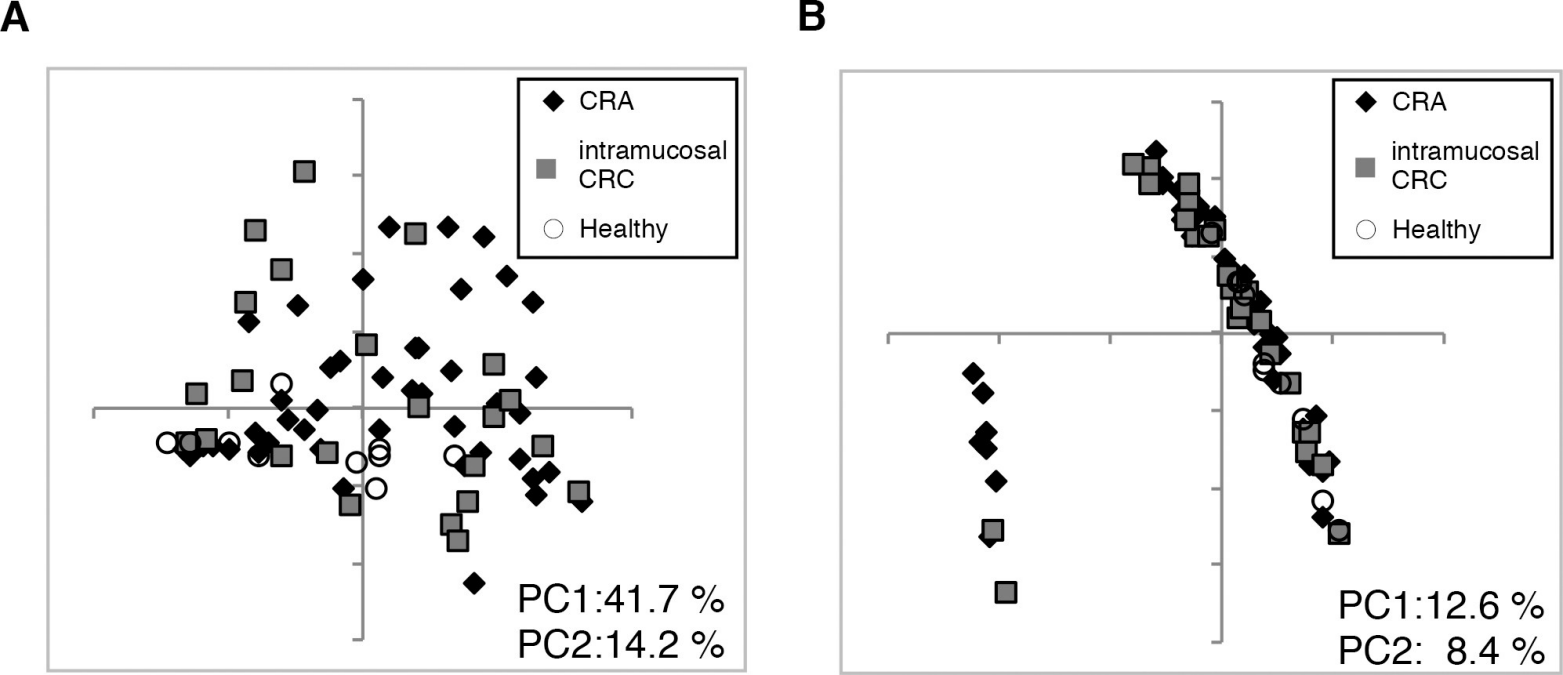
**Principal coordinates analysis (PCoA) based on (A) weighted and (B) unweighted UniFrac distance.** Diamond, CRA patient; Square, intramucosal CRC patient; Circle, healthy subject.

A total of 740,273 high-quality paired sequences were obtained from the 81 samples, with a mean of 9,139 ± 3,185. We first calculated UniFrac distances to determine the similarity between microbial communities. Weighted UniFrac PCoA (principal coordinate analysis) indicated that no distinct aggregation was observed between CRA and intramucosal CRC patients, whereas healthy subjects were found to be aggregated on the lower side (Fig 1A), suggesting a difference in gut microbiota composition between these patients and healthy subjects. Two distinct aggregations were observed in the data of Unweighted UniFrac PCoA; however, the subject in each group was not biased (Fig 1B), suggesting that the balance of gut microbes is more important than the existence of certain bacterial members in the CRA or intramucosal CRC patient gut. The Shannon index demonstrated a significant difference between CRA patients and healthy subjects (*p* = 0.019, S2 Fig). Moreover, there was no statistically significant difference in the Shannon index between patients with CRC and healthy subjects (*p* = 0.068, S2 Fig).

### LEfSe analysis and LDA based on OTUs characterize microbiomes in patients with CRA and healthy subjects

**Fig 2:**
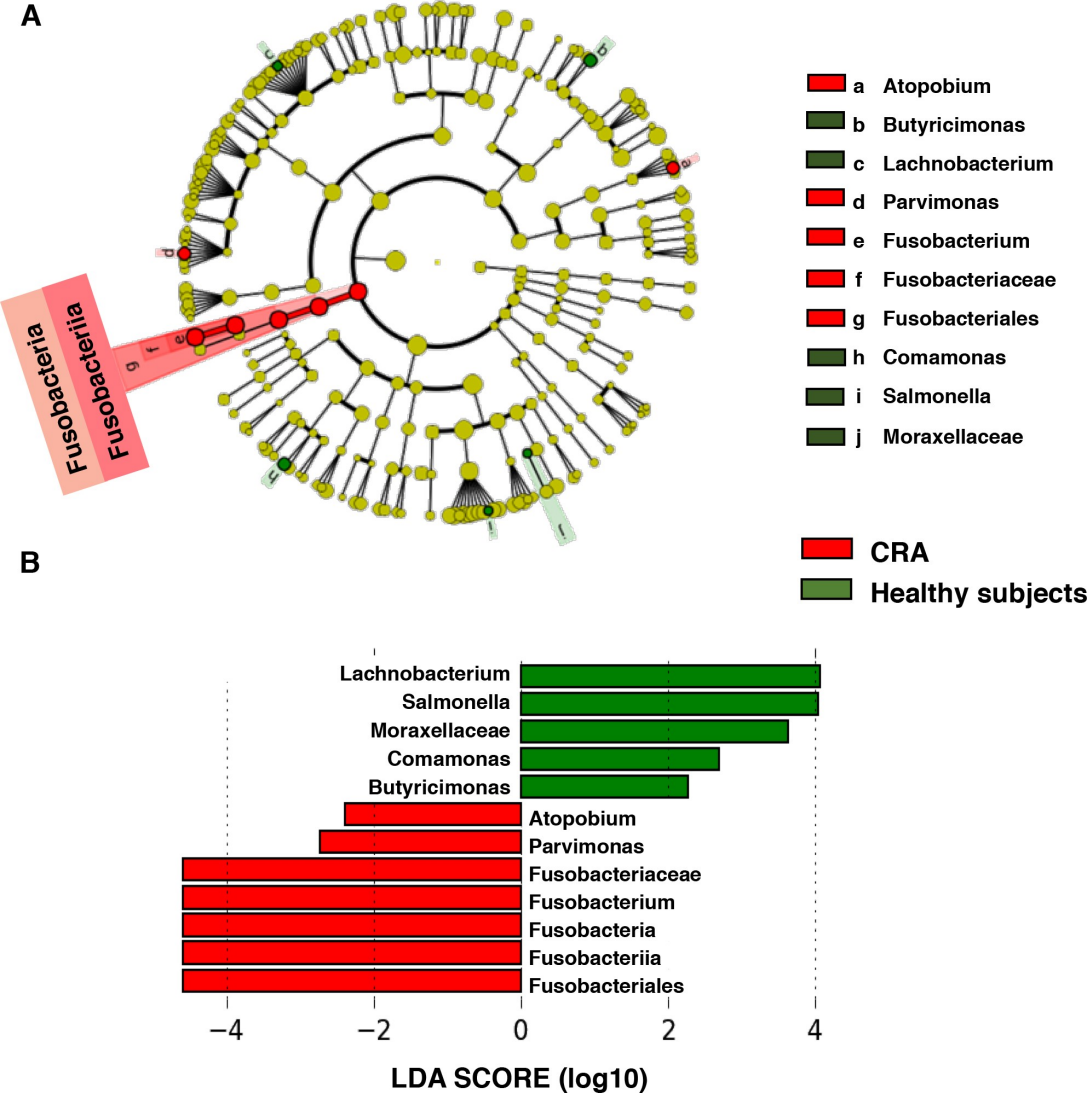
Characterization of microbiomes in CRA patients and healthy subjects by LEfSe analysis and LDA. (A) Taxonomic representation of statistically and biologically consistent differences in CRA and healthy subjects. (B) Histogram of the LDA scores (log10) computed for features with differential abundance in CRA patients and healthy subjects.

We then performed LEfSe analysis to compare the estimated phylotypes of patients with CRA and healthy microbiotas. The gut microbial communities in patients with CRA were diverse compared to those in healthy subjects. The results indicated differences in the phylogenetic distributions of the microbiotas of patients with CRA and those of healthy subjects at the OTU level (Fig 2A). A histogram of the LDA scores was computed for features that showed differential abundance between healthy subjects and CRA patients. The LDA scores indicated that the relative abundances of *Fusobacterium*, *Parvimonas*, and *Atopobium* were much more enriched in patients with CRA than in healthy subjects (Fig 2B). The most differentially abundant bacterial taxon in patients with CRA was *Fusobacterium* spp. (LDA score [log 10] > 3), whereas the healthy microbiome was characterized by a preponderance of *Lachnobacterium*, *Salmonella*, and Moraxellaceae (LDA score [log10] > 3) (Fig 2B).

### Bacteria whose relative abundances differ significantly between patients with CRA and healthy subjects

**Fig 3:**
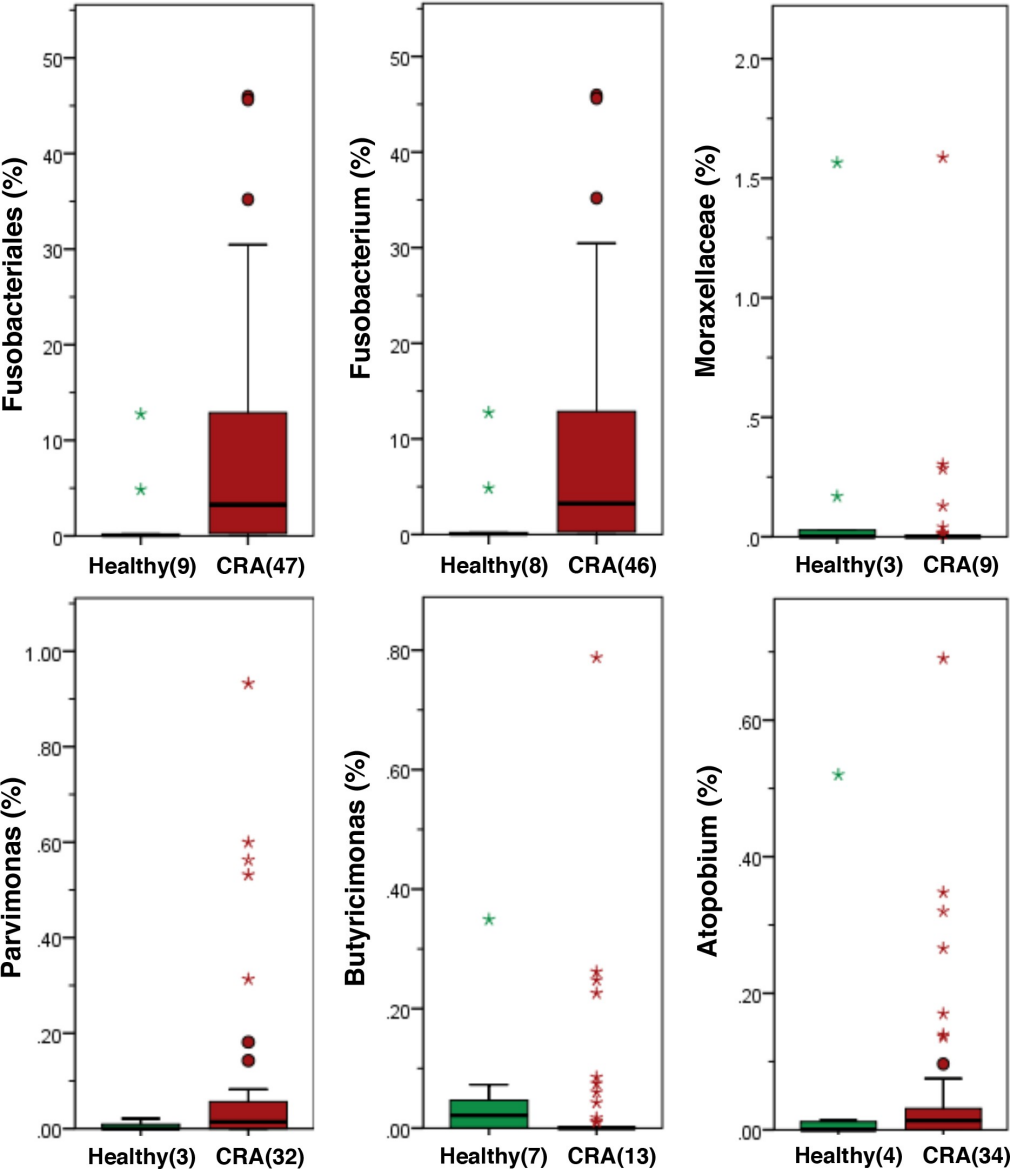
Histogram of the gut microbiota relative abundances in CRA patients and healthy subjects. The relative abundances of the groups of bacteria displayed significant differences between CRA patients and healthy subjects (LDA score [log 10]>3) (p < 0.001). Data are presented as the mean ± SE. Numbers in parentheses indicate the number of subjects positive for a given group of bacteria.

Among the bacteria whose relative abundances differed significantly between patients with CRA and healthy subjects, both Fusobacteriales and *Fusobacterium* were mainly detected in patients with CRA (Fig 3). The relative abundances of Moraxellaceae, *Parvimonas*, *Butyricimonas*, *Atopobium*, *Comamonas*, *Lachnobacterium*, and *Salmonella* were also significantly different between patients with CRA and healthy subjects (p < 0.001); however, the percentage of these bacteria relative to all bacteria was extremely small (less than 1%). Both Fusobacteriales and *Fusobacterium* were therefore mainly associated with CRA patients, in contrast to healthy subjects.

### Characterization of the microbiomes of patients with intramucosal CRC and healthy subjects by LEfSe analysis and LDA based on OTUs

**Fig 4:**
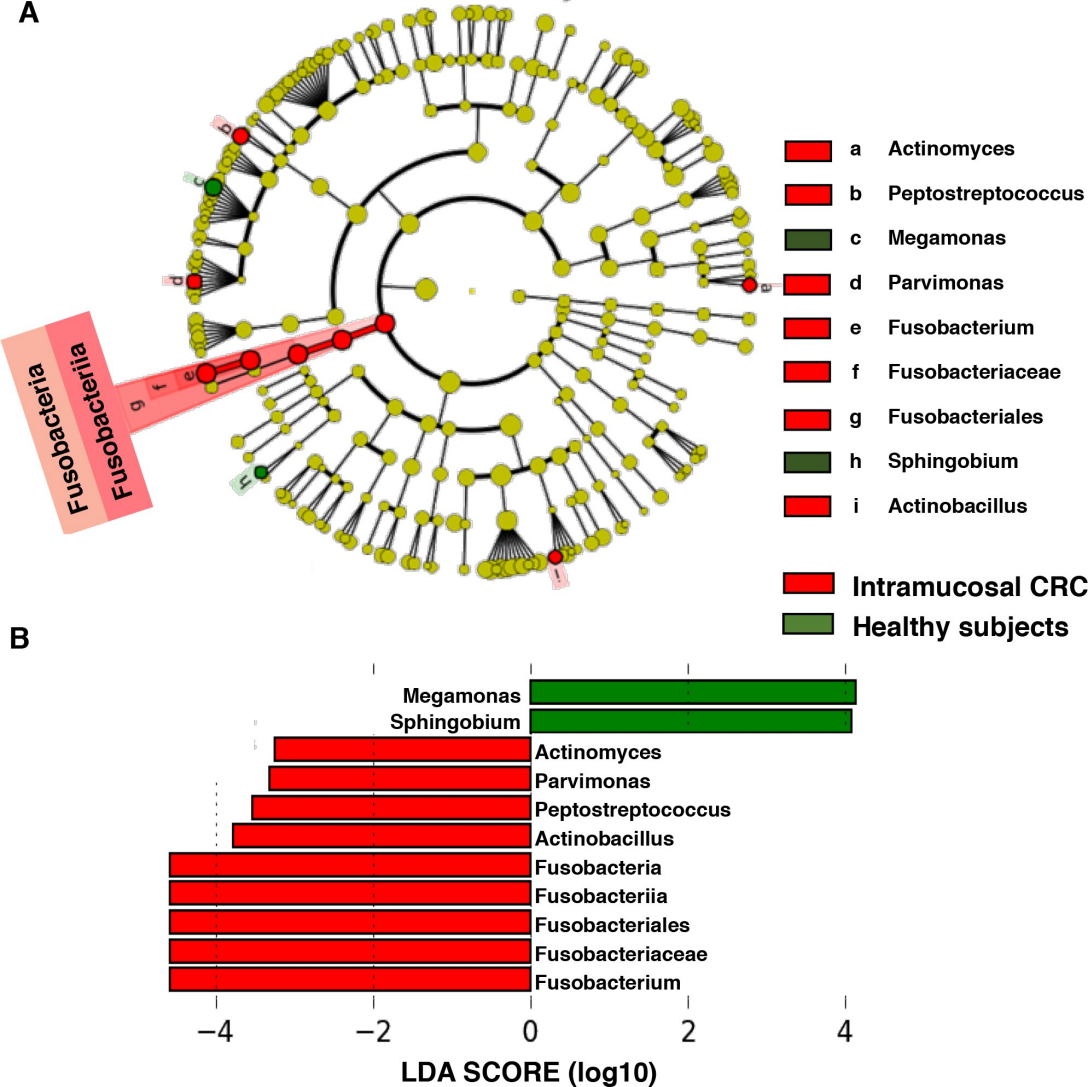
Characterization of microbiomes in intramucosal CRC patients and healthy subjects. (A) Taxonomic representation of statistically and biologically consistent differences between healthy subjects and intramucosal CRC patients. (B) Histogram of the LDA scores (log10) computed for features differentially abundant in CRC patients and healthy subjects.

LDA scores showed significant bacterial differences between healthy subjects and patients with intramucosal CRC (Fig 4A). The intramucosal CRC microbiome was characterized by a preponderance of *Fusobacterium*, *Actinobacillus*, *Peptostreptococcus*, *Parvimonas*, and *Actinomyces* (LDA score [log10] > 3), whereas the healthy microbiome was characterized by a preponderance of *Megamonas* and *Sphingobium* (LDA score [log10] > 3) (Fig 4B).

### Bacteria with relative abundances that significantly differ between patients with intramucosal CRC and healthy subjects

**Fig 5:**
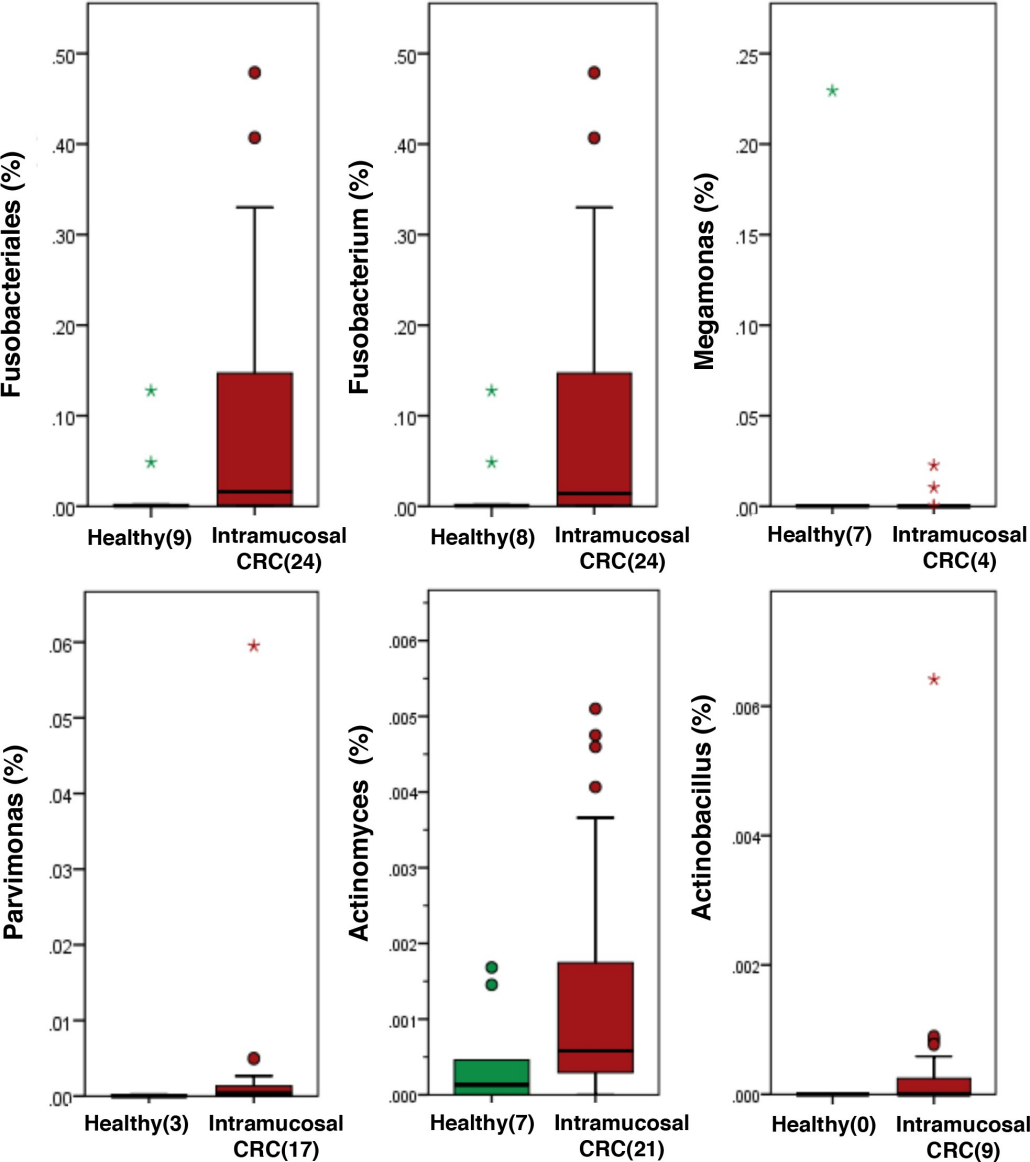
Histogram of the gut microbiota relative abundances in intramucosal CRC patients and healthy subjects. Relative abundances of the groups of bacteria that displayed significant differences between patients with intramucosal CRC and healthy subjects (LDA score [log 10]>3) (p < 0.001). Data are presented as the mean ± SE. Numbers in parentheses indicate the number of subjects positive for a given group of bacteria.

The relative abundances of bacteria significantly differed between patients with intramucosal CRC and healthy subjects (Fig 5). Although the percentages of *Megamonas*, *Peptostreptococcus*, *Parvimonas*, *Actinomyces*, *Actinobacillus*, and *Sphingobium* in colonoscopy aspirates were significantly different between patients with intramucosal CRC and healthy subjects (p < 0.001), these bacteria were present in extremely small populations (less than 1%). The intramucosal CRC microbiome was also mainly characterized by a preponderance of Fusobacteriales and *Fusobacterium* compared to healthy subjects (p < 0.001) (Fig 5).

### Characterization of *Fusobacterium* spp. in patients with CRA and healthy subjects

**Fig 6:**
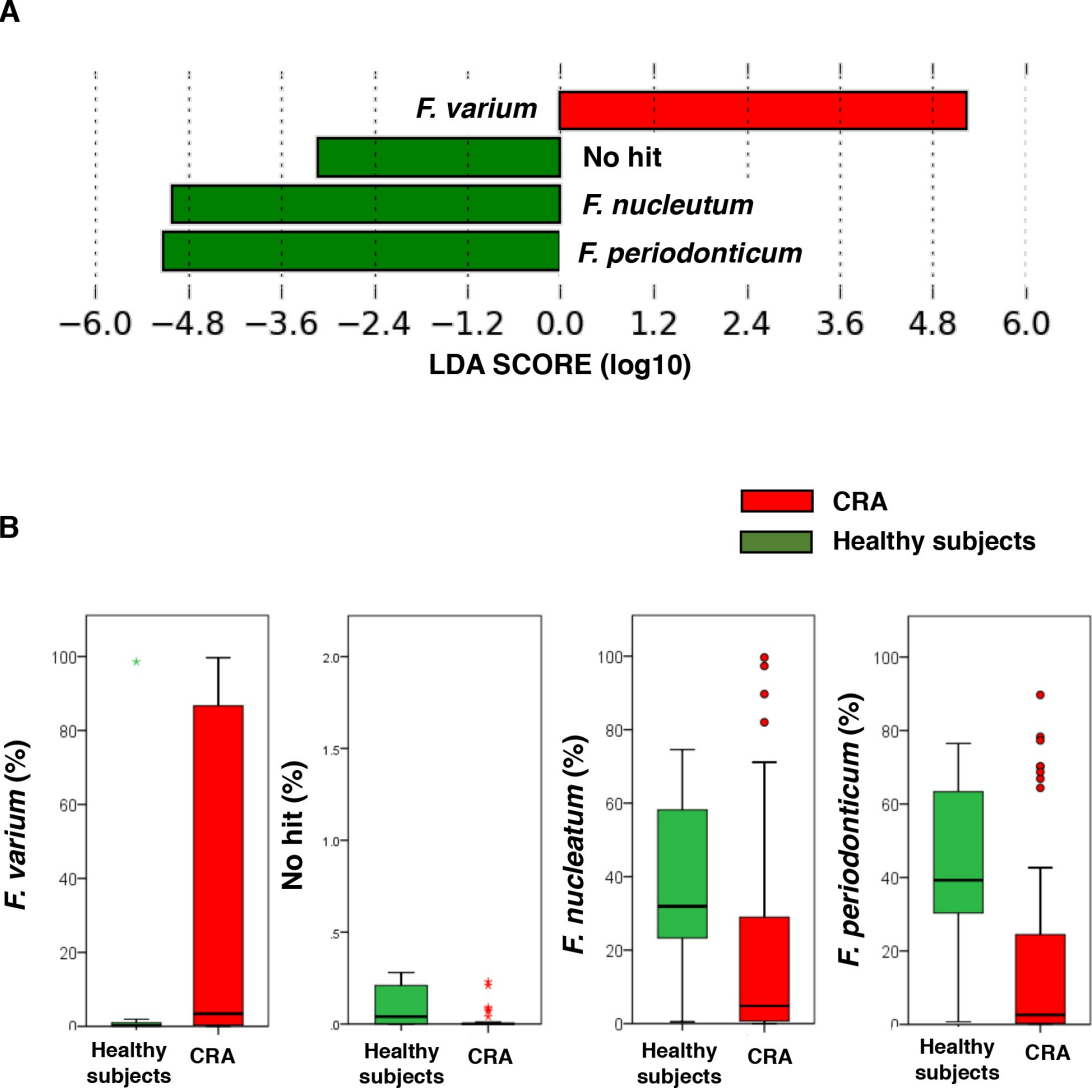
Characterization of *Fusobacterium* spp. in CRA patients and healthy subjects. (A) Histogram of the LDA scores (log10) computed for *Fusobacterium* spp. features that were differentially abundant in CRA and healthy subjects. (B) Relative abundances of the groups of *Fusobacterium* spp. that displayed significant differences between CRA and healthy subjects (LDA score [log 10]>3) (p < 0.001). Data are presented as the mean ± SE. No hit (%) indicates that there was no information on the components in the database.

*F*. *nucleatum* and *F*. *periodonticum* were detected in approximately 60% of healthy subjects, significantly more than in patients with CRA (Fig 6). Strikingly, *F*. *varium*, appearing in approximately 80% of CRA patients, could significantly distinguish CRA patients from healthy subjects (Fig 6A and 6B).

### Characterization of *Fusobacterium* spp. in patients with intramucosal CRC and healthy subjects

**Fig 7:**
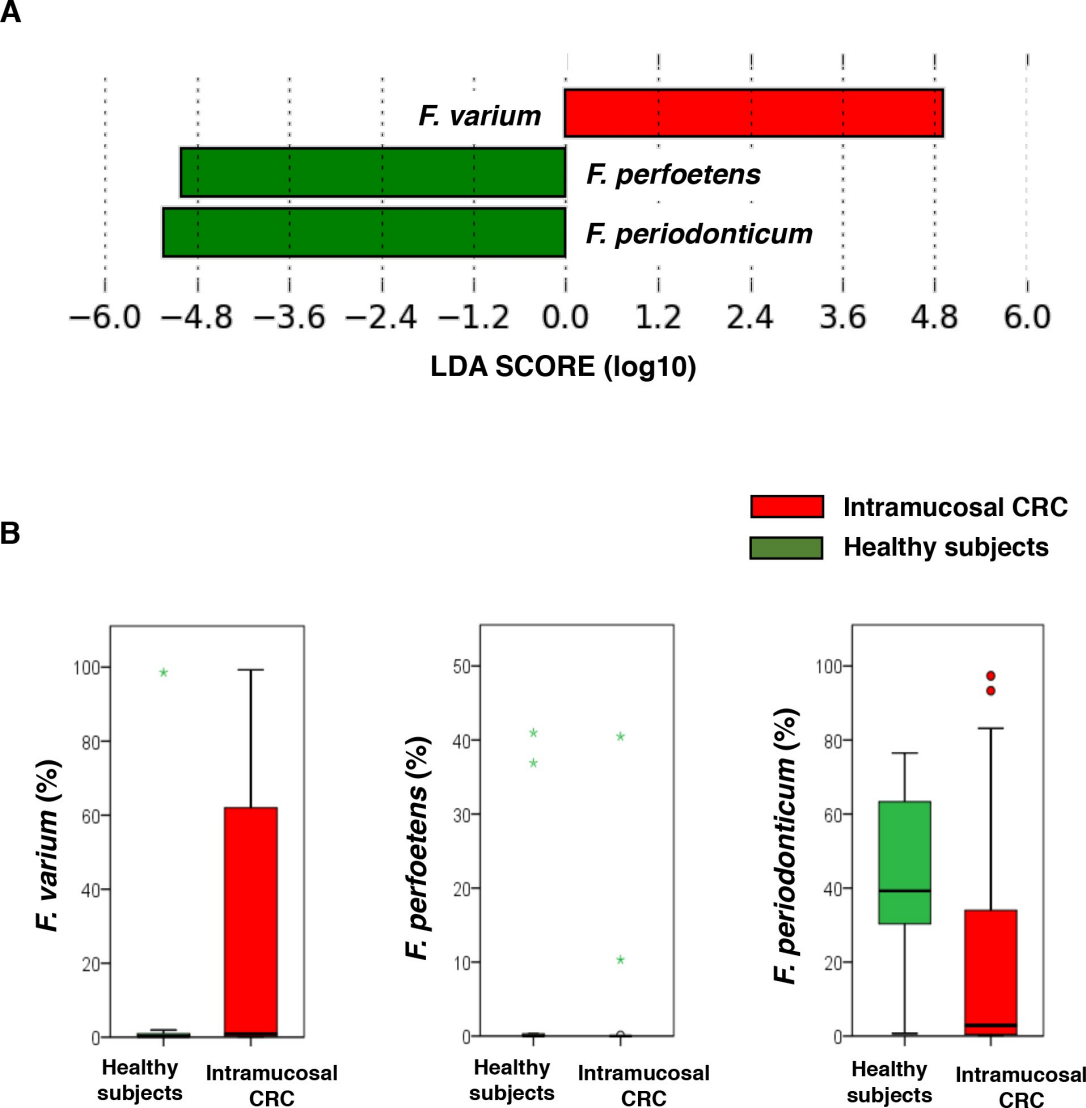
Characterization of *Fusobacterium* spp. in intramucosal CRC patients and healthy subjects. (A) Histogram of the LDA scores (log10) computed for *Fusobacterium* spp. Features that were differentially abundant in intramucosal CRC and healthy subjects. (B) Relative abundances of the groups of *Fusobacterium* spp. displaying significant differences between intramucosal CRC and healthy subjects. (LDA score [log 10]>3) (p < 0.001). Data are presented as the mean ± SE.

LEfSe analysis showed that *F*. *varium* was significantly more abundant in intramucosal CRC patients than in healthy subjects (Fig 7A and 7B). Moreover, approximately 60% of intramucosal CRC patients were *F*. *varium*-positive, whereas *F*. *perfoetens* and *F*. *periodonticum* were significantly detected in healthy subjects.

### Characterization of microbiomes in patients with CRA and intramucosal CRC by LEfSe analysis and LDA based on OTUs

**Fig 8:**
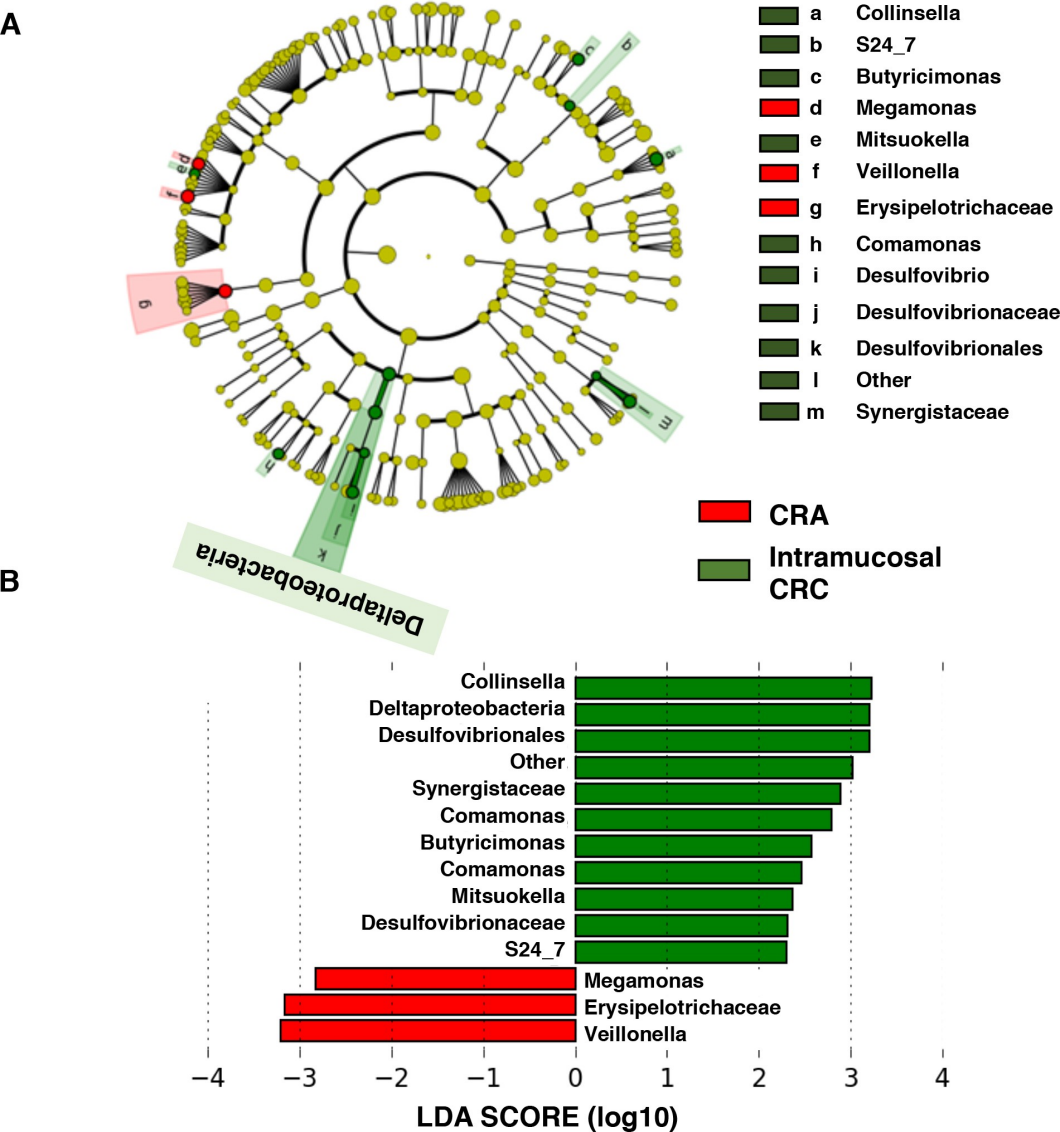
Characterization of microbiomes in patients with CRA and intramucosal CRC by LEfSe analysis and LDA. (A) Taxonomic representation of statistically and biologically consistent differences in patients with CRA and intramucosal CRC. (B) Histogram of the LDA scores (log10) computed for features that were differentially abundant in patients with CRA and intramucosal CRC.

Next, to compare the characteristic microbiomes in patients with CRA and intramucosal CRC, we performed LEfSe analysis. The LDA scores showed significant bacterial differences between patients with CRA and intramucosal CRC (Fig 8A). The intramucosal CRC microbiome was characterized by a preponderance of *Collinsella*, *Deltaproteobacteria*, and *Desulfovibrionales* (LDA score [log10] > 3), whereas the CRA microbiome was characterized by a preponderance of *Erysipelotrichaceae* and *Veillonella* (LDA score [log10] > 3) (Fig 8B). The LDA scores of both Fusobacteriales and *Fusobacterium* were not different between CRA and intramucosal CRC.

### Bacteria whose relative abundances differ significantly between patients with CRA and intramucosal CRC

**Fig 9:**
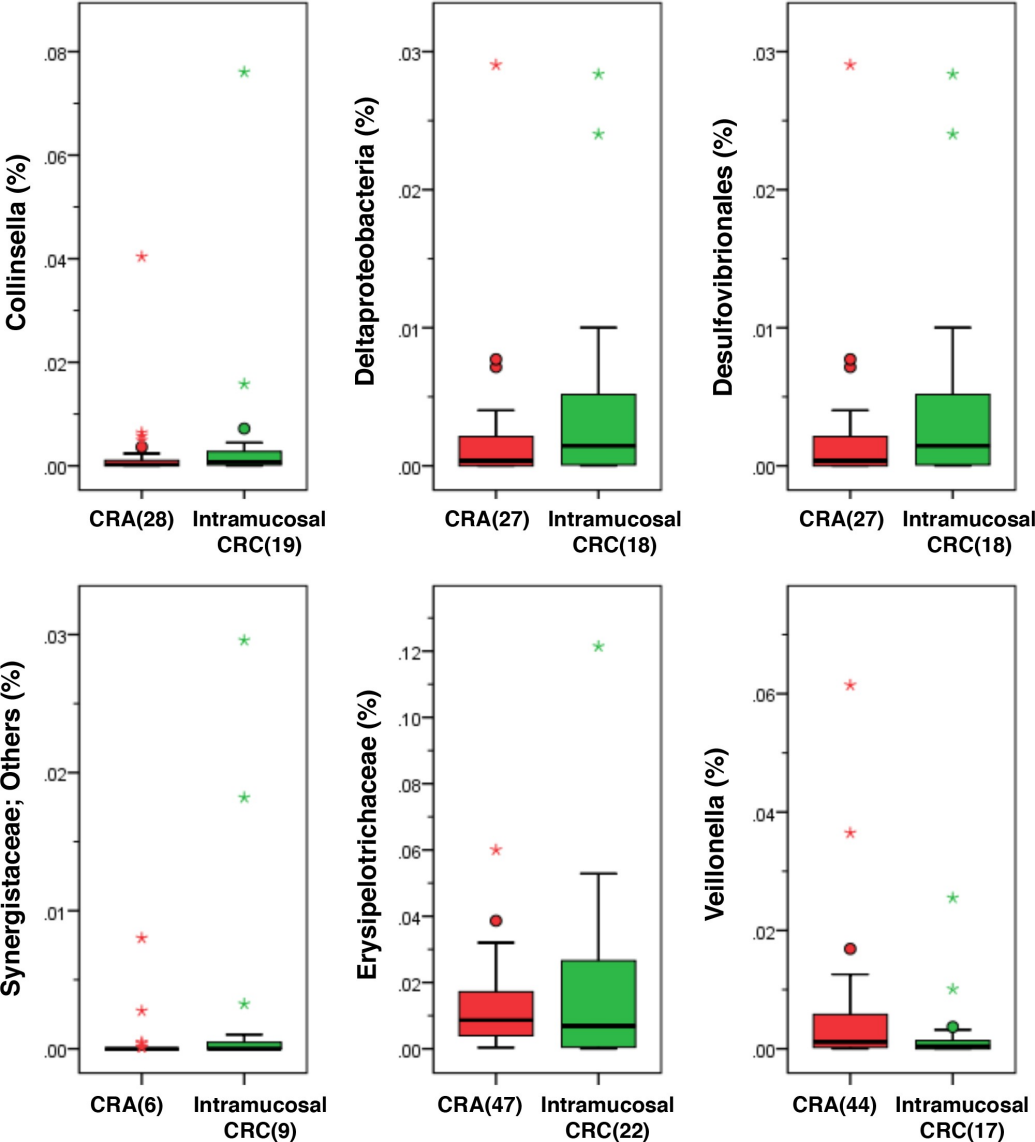
Histogram of the gut microbiota relative abundances in patients with CRA and intramucosal CRC. Relative abundances of the groups of bacteria that displayed significant differences between CRA patients and healthy subjects (LDA score [log 10]>3) (p < 0.001). Data are presented as the mean ± SE. Numbers in parentheses indicate the number of subjects positive for a given group of bacteria.

The relative abundances of *Collinsella*, *Deltaproteobacteria*, *Desulfovibrionales*, *Synergistaceae*; Other, *Erysipelotrichaceae*, and *Veillonella* were significantly different between patients with CRA and intramucosal CRC (p < 0.001); however, the percentage of these bacteria relative to all bacteria was extremely small (less than 1%) (Fig 9).

## Discussion

The present study has demonstrated for the first time that *F*. *varium* may be associated with the development of CRA as well as intramucosal CRC, as analyzed by a metagenomic approach using next-generation sequencing (NGS).

Previous studies have found the enrichment of *F*. *nucleatum* to be associated with advanced CRC from frozen mucosal tissues [4, 5] and fecal samples [25]. In clinical situations, analyses of the gut microbiota have limitations, including small frozen sample sizes and the use of fecal samples and undefined tissue sampling sites. However, colonoscopic technologies are suitable for diagnosing colorectal neoplasia, and colonoscopy aspirates are easily obtained for diagnosis. As it has been reported that colonoscopy aspirates can serve as a substitute for the gut microbiota in tissues [15], we analyzed the gut microbiota in a Japanese population using colonoscopy aspirates. We detected an abundance of *Fusobacterium* spp. in the colonoscopy aspirates of CRA or intramucosal CRC patients. Moreover, *F*. *varium* accounted for approximately 80% of the *Fusobacterium* spp. from CRA patients and 60% from intramucosal CRC patients. There was no difference in the *Fusobacterium* spp. between CRA and intramucosal CRC. *F*. *nucleatum* accounted for approximately 60% of the *Fusobacterium* spp. population in healthy controls, which was significantly higher than that in CRA patients. Previous studies have also reported that *F*. *nucleatum* can be detected in the stool and colon mucosa of healthy controls as well as CRC patients [26]. *F*. *varium* may show specificity for CRA and intramucosal CRC compared to healthy subjects. High copy numbers of *F*. *nucleatum* in advanced CRC tissues were associated with late stages and worse prognoses in a Japanese population [26]. *F*. *nucleatum* may mainly play a role in the progression of advanced CRC. The gut microbiome is characterized by dietary habits (rich in red meat relative to vegetables and fruits), living environments, and metabolic levels in different human races [27]. *F*. *nucleatum* was more abundant in patients with advanced CRC from Spain than in those from the United States and Vietnam. Moreover, a recent report demonstrated that the frequency of *F*. *nucleatum* positivity in Japanese patients with advanced CRC was much lower than that in similar patients in United States cohort studies [7]. The patient populations may be at least part of the reason why *F*. *varium* but not *F*. *nucleatum* was found to be enriched in CRA or intramucosal CRC patients compared to healthy subjects. Our current data, which indicate greater abundance of *F*. *varium* in patients with CRA and intramucosal CRC than in healthy subjects in a Japanese population, suggest that *Fusobacterium* spp. colonization may vary regionally. However, the relationship between the gut microbiota and human races is still unknown. Moreover, the different sample types analyzed from colonoscopy aspirates in this study should also be associated with the different results. In this study, we did not analyze advanced CRC; therefore, the association of *F*. *varium* with advanced CRC may also be possible. Future studies are needed to analyze the gut microbiota from colonoscopic aspirates in adenoma, intramucosal CRC, and advanced CRC in the same sets of experiments.

*F*. *varium* can invade colonic epithelial cells, activating early intracellular signaling systems to trigger host inflammatory reactions [11]. Adhering to the colonic mucosa is essential for the gut microbiota to deliver specific oncogenic molecules into the colonic epithelial cells, which results in inflammation and oncogenic signaling in the colonic epithelial cells [28]. We recently reported the complete genome sequence of *F*. *varium* Fv113-g1, which had been isolated from a patient with UC. Fv113-g1 possessed many accessary pangenome sequences with noteworthy multiple virulence factors, including FadA, in contrast to *F*. *nucleatum* [9]. Kasper’s group also reported that *F*. *varium* suppresses *Reg3* to avoid death induced by antimicrobial peptides (AMPs), promoting intestinal barrier breaks [29]. These results suggest that *F*. *varium* is pathogenic in human colorectal tumors. However, the association of *F*. *varium* with neoplasm proliferation is unknown. In this study, we analyzed not tissue samples but colonoscopic aspirates. Future studies of interactions between *F*. *varium* and neoplasm proliferation are needed to assess the possible association of *F*. *varium* with the initiation and/or progression of colorectal carcinogenesis. Outgrowth of *Fusobacterium* spp. such as *F*. *varium* and *F*. *nucleatum* following dysbiosis may be a more important cause of colorectal carcinogenesis. The human gut microbiota may contribute to the etiology of CRC, not only via the procarcinogenic activities of specific pathogens such as *Fusobacterium* spp. but also via the influence of the wider microbe-induced networks for metabolic pathway aberrations [1]. Mechanisms causing *Fusobacterium* abundance and oncogenic properties in other gastrointestinal tract tumors and pancreatobiliary tumors remain unknown.

The complex interactions of hosts and their gut microbiotas also contribute to the pathogenesis of CRC. In human host environments, *F*. *nucleatum* modulates tumor-associated inflammation, such as that involving regulatory T cells (Tregs), in the tumor microenvironment with consequences for the inhibition or promotion of CRC biology [24]. Moreover, *F*. *nucleatum* expands myeloid-derived immune cells, which inhibit T cell proliferation and induce T cell apoptosis in patients with CRC, resulting in immunosuppression [7]. Not only *F*. *nucleatum* but also *F*. *varium* may influence inflammation of the colorectum. A recent report indicated that *F*. *varium* decreased both CD4+ and CD8+ populations more strongly and caused a higher frequency of colonic CD4-CD8-T cells than any other microbe [30]. The mechanisms of interactions between hosts and *F*. *varium* and *F*. *nucleatum* should be further investigated.

As enhanced abundances of *Fusobacterium* spp. such as *F*. *varium* and *F*. *nucleatum* may also be associated with some patients with CRC, *Fusobacterium* spp. detection in colonoscopy aspirates may be sufficient for identifying patients with increased risks for CRA or CRC [4, 5]. Moreover, high levels of serum anti-*Fusobacterium* spp. antibodies are also a potential biomarker for CRC diagnosis [31]. Targeting *Fusobacterium* spp. with antimicrobial interventions may be a potential treatment or method of prevention for patients with *Fusobacterium*-associated CRC. Treatment of a mouse model bearing colon cancer with the antibiotic metronidazole certainly reduced *Fusobacterium* amounts, the proliferating activity of cancer cells, and overall tumor growth [32]. Further investigation of antimicrobial interventions as a potential treatment for patients with *Fusobacterium*-associated CRC is also interesting.

## Supporting information

S1 Fig Primer design based on the internal transcribed spacer (ITS) region of the Fusobacterium group. Open arrows indicate the position of a designed primer set. The attached and underlined regions of the primers indicate the binding site for the barcode primer for the 2^nd^ PCR. The numbers at the top of the sequences indicate the bases according to the nucleotide sequence of the ITS region in *Fusobacterium nucleatum* subsp. *nucleatum* ATCC255836^T^. A dot indicates a conserved sequence.
(TIF)

S2 Fig Shannon diversity index as alpha diversity of the gut microbiota. The bold line indicates the average Shannon diversity index in each group.
(TIF)

S1 Table Information of samples for V3-4 region of 16s rRNA gene sequencing. (DOCX)

S2 Table Information of samples for ITS region of Fusobacterium spp. sequencing. (DOCX)

## References

[1] Louis P, Hold GL, Flint HJ. The gut microbiota, bacterial metabolites and colorectal cancer. Nature reviews Microbiology. 2014;12(10):661–72. Epub 2014/09/10. 10.1038/nrmicro3344 . 25198138

[2] Keku TO, Dulal S, Deveaux A, Jovov B, Han X. The gastrointestinal microbiota and colorectal cancer. American journal of physiology Gastrointestinal and liver physiology. 2015;308(5):G351–63. Epub 2014/12/30. 10.1152/ajpgi.00360.2012 25540232 PMC4346754

[3] Arthur JC, Perez-Chanona E, Muhlbauer M, Tomkovich S, Uronis JM, Fan TJ, et al Intestinal inflammation targets cancer-inducing activity of the microbiota. Science (New York, NY). 2012;338(6103):120–3. Epub 2012/08/21. 10.1126/science.1224820 22903521 PMC3645302

[4] Castellarin M, Warren RL, Freeman JD, Dreolini L, Krzywinski M, Strauss J, et al Fusobacterium nucleatum infection is prevalent in human colorectal carcinoma. Genome research. 2012;22(2):299–306. Epub 2011/10/20. 10.1101/gr.126516.111 22009989 PMC3266037

[5] Kostic AD, Gevers D, Pedamallu CS, Michaud M, Duke F, Earl AM, et al Genomic analysis identifies association of Fusobacterium with colorectal carcinoma. Genome research. 2012;22(2):292–8. Epub 2011/10/20. 10.1101/gr.126573.111 22009990 PMC3266036

[6] Yang Y, Weng W, Peng J, Hong L, Yang L, Toiyama Y, et al Fusobacterium nucleatum increases proliferation of colorectal cancer cells and tumor development in mice by activating Toll-like receptor 4 signaling to nuclear factor-kappaB, and up-regulating expression of microRNA-21. Gastroenterology. 2017;152(4):851–66.e24. Epub 2016/11/24. 10.1053/j.gastro.2016.11.018 . 27876571 PMC5555435

[7] Mima K, Sukawa Y, Nishihara R, Qian ZR, Yamauchi M, Inamura K, et al Fusobacterium nucleatum and T cells in colorectal carcinoma. JAMA oncology. 2015;1(5):653–61. Epub 2015/07/17. 10.1001/jamaoncol.2015.1377 26181352 PMC4537376

[8] Sears CL, Garrett WS. Microbes, microbiota, and colon cancer. Cell host & microbe. 2014;15(3):317–28. Epub 2014/03/19. 10.1016/j.chom.2014.02.007 24629338 PMC4003880

[9] Sekizuka T, Ogasawara Y, Ohkusa T, Kuroda M. Characterization of Fusobacterium varium Fv113-g1 isolated from a patient with ulcerative colitis based on complete genome sequence and transcriptome analysis. PloS one. 2017;12(12):e0189319 Epub 2017/12/08. 10.1371/journal.pone.0189319 29216329 PMC5720691

[10] Allen-Vercoe E, Strauss J, Chadee K. Fusobacterium nucleatum: an emerging gut pathogen? Gut microbes. 2011;2(5):294–8. Epub 2011/11/10. 10.4161/gmic.2.5.18603 . 22067936

[11] Ohkusa T, Yoshida T, Sato N, Watanabe S, Tajiri H, Okayasu I. Commensal bacteria can enter colonic epithelial cells and induce proinflammatory cytokine secretion: a possible pathogenic mechanism of ulcerative colitis. Journal of medical microbiology. 2009;58(Pt 5):535–45. Epub 2009/04/17. 10.1099/jmm.0.005801-0 19369513 PMC2887547

[12] Oh JK, Weiderpass E. Infection and cancer: global distribution and burden of diseases. Annals of global health. 2014;80(5):384–92. Epub 2014/12/17. 10.1016/j.aogh.2014.09.013 . 25512154

[13] Nakatsu G, Li X, Zhou H, Sheng J, Wong SH, Wu WK, et al Gut mucosal microbiome across stages of colorectal carcinogenesis. Nature communications. 2015;6:8727 Epub 2015/10/31. 10.1038/ncomms9727 26515465 PMC4640069

[14] Rubinstein MR, Wang X, Liu W, Hao Y, Cai G, Han YW. Fusobacterium nucleatum promotes colorectal carcinogenesis by modulating E-cadherin/beta-catenin signaling via its FadA adhesin. Cell host & microbe. 2013;14(2):195–206. Epub 2013/08/21. 10.1016/j.chom.2013.07.012 23954158 PMC3770529

[15] Keren N, Naftali T, Kovacs A, Konikoff FM, Gophna U. Can colonoscopy aspirates be a substitute for fecal samples in analyses of the intestinal microbiota? Bioscience of microbiota, food and health. 2012;31(3):71–6. Epub 2012/01/01. 10.12938/bmfh.31.71 24936352 PMC4034279

[16] Hari DM, Leung AM, Lee JH, Sim MS, Vuong B, Chiu CG, et al AJCC Cancer Staging Manual 7th edition criteria for colon cancer: do the complex modifications improve prognostic assessment? Journal of the American College of Surgeons. 2013;217(2):181–90. Epub 2013/06/19. 10.1016/j.jamcollsurg.2013.04.018 23768788 PMC4657944

[17] Edge SB, Byrd SR, Compton CC, Fritz AG, Greene FL, Trottl I, A. AJCC cancer staging manual. 7th edition Springer-Verlag; New York (NY) 2010:143–64.

[18] Kim SW, Suda W, Kim S, Oshima K, Fukuda S, Ohno H, et al Robustness of gut microbiota of healthy adults in response to probiotic intervention revealed by high-throughput pyrosequencing. DNA research: an international journal for rapid publication of reports on genes and genomes. 2013;20(3):241–53. Epub 2013/04/11. 10.1093/dnares/dst006 23571675 PMC3686430

[19] Odamaki T, Bottacini F, Kato K, Mitsuyama E, Yoshida K, Horigome A, et al Genomic diversity and distribution of Bifidobacterium longum subsp. longum across the human lifespan. Scientific reports. 2018;8(1):85 Epub 2018/01/10. 10.1038/s41598-017-18391-x . 29311585 PMC5758520

[20] Kuczynski J, Stombaugh J, Walters WA, Gonzalez A, Caporaso JG, Knight R. Using QIIME to analyze 16S rRNA gene sequences from microbial communities. Current protocols in microbiology. 2012;Chapter 1:Unit 1E.5. Epub 2012/11/28. 10.1002/9780471729259.mc01e05s27 23184592 PMC4477843

[21] Caporaso JG, Kuczynski J, Stombaugh J, Bittinger K, Bushman FD, Costello EK, et al QIIME allows analysis of high-throughput community sequencing data. Nature methods. 2010;7(5):335–6. Epub 2010/04/13. 10.1038/nmeth.f.303 20383131 PMC3156573

[22] Barberan A, Dunn RR, Reich BJ, Pacifici K, Laber EB, Menninger HL, et al The ecology of microscopic life in household dust. Proceedings Biological sciences. 2015;282(1814). Epub 2015/08/28. 10.1098/rspb.2015.1139 26311665 PMC4571696

[23] Segata N, Izard J, Waldron L, Gevers D, Miropolsky L, Garrett WS, et al Metagenomic biomarker discovery and explanation. Genome biology. 2011;12(6):R60 Epub 2011/06/28. 10.1186/gb-2011-12-6-r60 21702898 PMC3218848

[24] de la Cuesta-Zuluaga J, Mueller NT, Corrales-Agudelo V, Velasquez-Mejia EP, Carmona JA, Abad JM, et al Metformin Is associated with higher relative abundance of mucin-degrading akkermansia muciniphila and several short-chain fatty acid-producing microbiota in the gut. Diabetes care. 2017;40(1):54–62. Epub 2016/12/22. 10.2337/dc16-1324 . 27999002

[25] Ahn J, Sinha R, Pei Z, Dominianni C, Wu J, Shi J, et al Human gut microbiome and risk for colorectal cancer. Journal of the National Cancer Institute. 2013;105(24):1907–11. Epub 2013/12/10. 10.1093/jnci/djt300 24316595 PMC3866154

[26] Yamaoka Y, Suehiro Y, Hashimoto S, Hoshida T, Fujimoto M, Watanabe M, et al Fusobacterium nucleatum as a prognostic marker of colorectal cancer in a Japanese population. Journal of gastroenterology. 2017 Epub 2017/08/22. 10.1007/s00535-017-1382-6 . 28823057

[27] Chen L, Zhang YH, Huang T, Cai YD. Gene expression profiling gut microbiota in different races of humans. Scientific reports. 2016;6:23075 Epub 2016/03/16. 10.1038/srep23075 26975620 PMC4791684

[28] Soler AP, Miller RD, Laughlin KV, Carp NZ, Klurfeld DM, Mullin JM. Increased tight junctional permeability is associated with the development of colon cancer. Carcinogenesis. 1999;20(8):1425–31. Epub 1999/07/30. . 10426787 10.1093/carcin/20.8.1425

[29] Geva-Zatorsky N, Sefik E, Kua L, Pasman L, Tan TG, Ortiz-Lopez A, et al Mining the human gut microbiota for immunomodulatory organisms. Cell. 2017;168(5):928–43.e11. Epub 2017/02/22. 10.1016/j.cell.2017.01.022 . 28215708 PMC7774263

[30] Saito T, Nishikawa H, Wada H, Nagano Y, Sugiyama D, Atarashi K, et al Two FOXP3(+)CD4(+) T cell subpopulations distinctly control the prognosis of colorectal cancers. Nature medicine. 2016;22(6):679–84. Epub 2016/04/26. 10.1038/nm.4086 . 27111280

[31] Wang HF, Li LF, Guo SH, Zeng QY, Ning F, Liu WL, et al Evaluation of antibody level against Fusobacterium nucleatum in the serological diagnosis of colorectal cancer. Scientific reports. 2016;6:33440 Epub 2016/09/30. 10.1038/srep33440 27678333 PMC5039407

[32] Bullman S, Pedamallu CS, Sicinska E, Clancy TE, Zhang X, Cai D, et al Analysis of Fusobacterium persistence and antibiotic response in colorectal cancer. Science (New York, NY). 2017;358(6369):1443–8. Epub 2017/11/25. 10.1126/science.aal5240 . 29170280 PMC5823247

