# Expression of Concern: Metagenomic analyses of the gut microbiota associated with colorectal adenoma

**DOI:** 10.1371/journal.pone.0280121

**Published:** 2023-01-11

**Authors:** The PLOS ONE Editors

The Funding Statement for this article [1] states that the study received funding from the Smoking Research Foundation, which according to [2] has received financial support from the tobacco industry. In light of this issue the *PLOS ONE* article [1] does not comply with the journal’s policy on Funding from Tobacco Companies [3] which was implemented in 2010.

Therefore, the *PLOS ONE* Editors issue this Expression of Concern.

In post-publication discussions the corresponding author stated that Smoking Research Foundation funding did not support this study and was reported in error.

We regret that this concern was not identified and addressed prior to the article’s [1] publication.
