# Supplementary figures and images for: Metagenomic analyses of the gut microbiota associated with colorectal adenoma
Source: PLoS One. 2019 Feb 22;14(2):e0212406. doi: 10.1371/journal.pone.0212406 (PMC6386391; doi:10.1371/journal.pone.0212406)

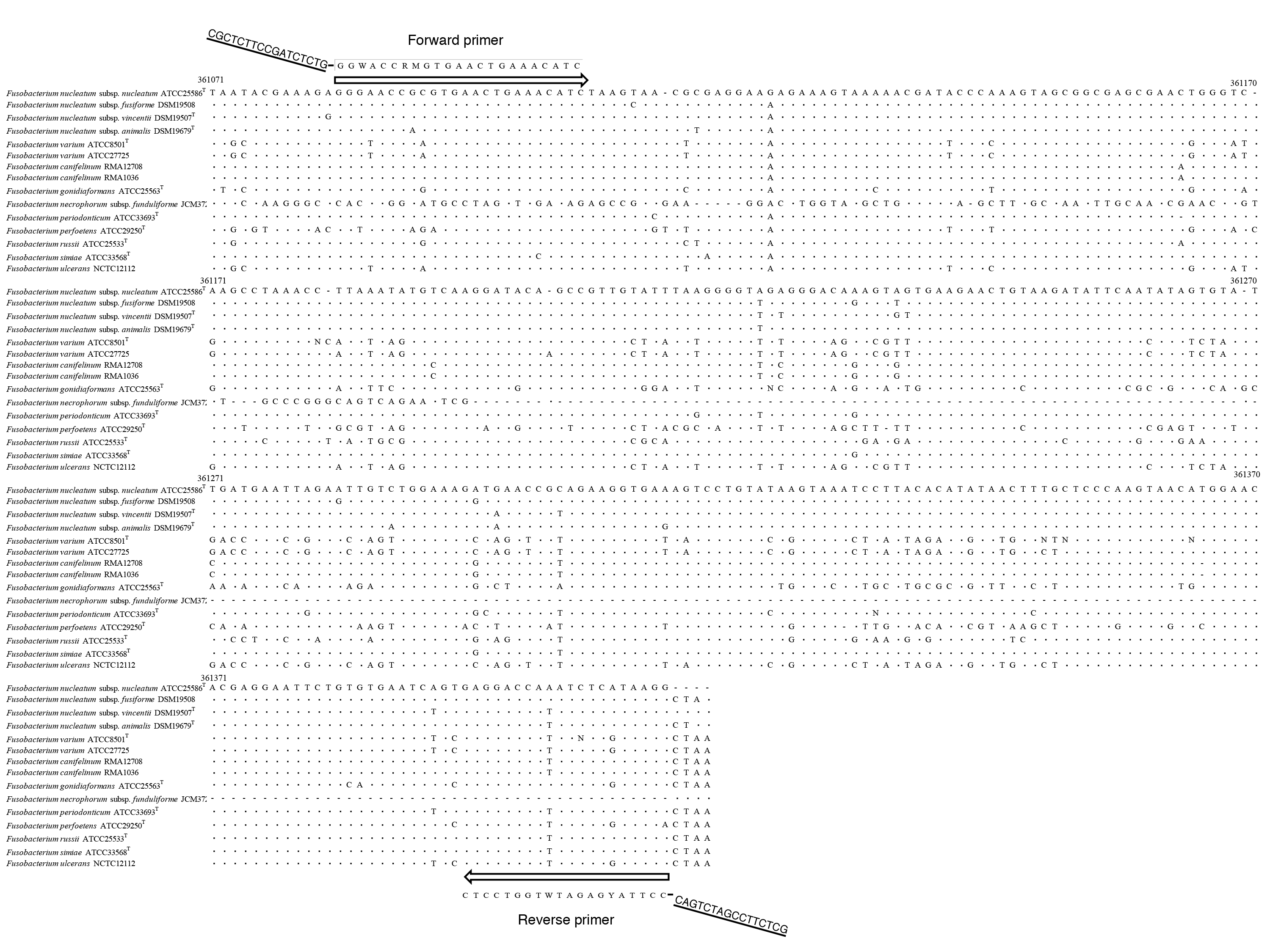

Supplement: S1 Fig — Open arrows indicate the position of a designed primer set. The attached and underlined regions of the primers indicate the binding site for the barcode primer for the 2nd PCR. The numbers at the top of the sequences indicate the bases according to the nucleotide sequence of the ITS region in Fusobacterium nucleatum subsp. nucleatum ATCC255836T. A dot indicates a conserved sequence. (TIF) [file pone.0212406.s001.tif]

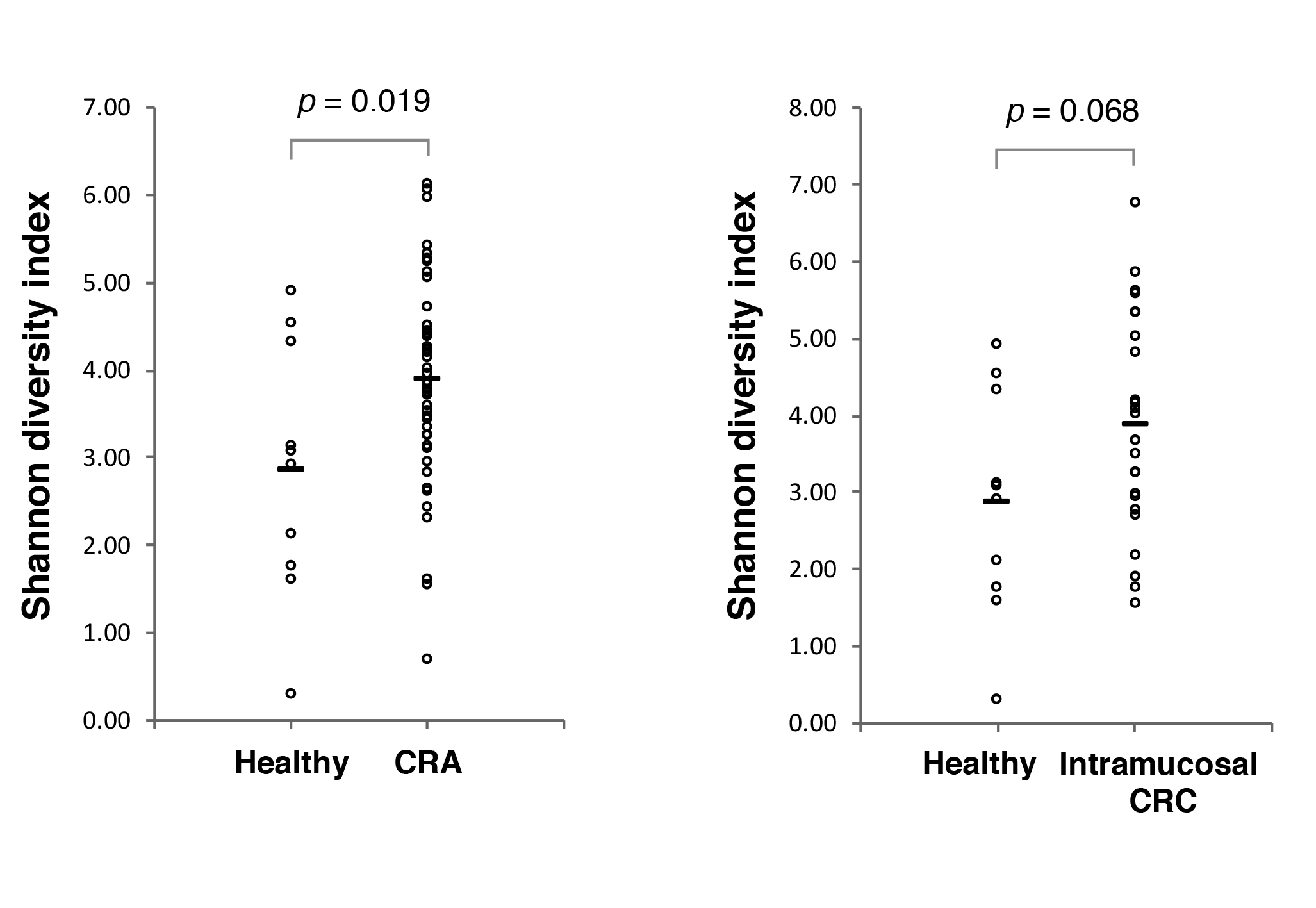

Supplement: S2 Fig — The bold line indicates the average Shannon diversity index in each group. (TIF) [file pone.0212406.s002.tif]
