# Supplementary material for: Metagenomic analyses of the gut microbiota associated with colorectal adenoma
Source: PLoS One. 2019 Feb 22;14(2):e0212406. doi: 10.1371/journal.pone.0212406 (PMC6386391; doi:10.1371/journal.pone.0212406)
Supplement: S1 Table — (DOCX) [file pone.0212406.s003.docx]

Supplementary Tale S1. Information of samples for V3-4 region of 16s rRNA gene sequencing

| Subject  (name of deposit data) | BioSample No. |
| --- | --- |
| Subject001 | SAMD00156360 |
| Subject002 | SAMD00156361 |
| Subject003 | SAMD00156362 |
| Subject004 | SAMD00156363 |
| Subject005 | SAMD00156364 |
| Subject006 | SAMD00156365 |
| Subject007 | SAMD00156366 |
| Subject008 | SAMD00156367 |
| Subject009 | SAMD00156368 |
| Subject010 | SAMD00156369 |
| Subject011 | SAMD00156370 |
| Subject012 | SAMD00156371 |
| Subject013 | SAMD00156372 |
| Subject014 | SAMD00156373 |
| Subject015 | SAMD00156374 |
| Subject016 | SAMD00156375 |
| Subject017 | SAMD00156376 |
| Subject018 | SAMD00156377 |
| Subject019 | SAMD00156378 |
| Subject020 | SAMD00156379 |
| Subject021 | SAMD00156380 |
| Subject022 | SAMD00156381 |
| Subject023 | SAMD00156382 |
| Subject024 | SAMD00156383 |
| Subject025 | SAMD00156384 |
| Subject026 | SAMD00156385 |
| Subject027 | SAMD00156386 |
| Subject028 | SAMD00156387 |
| Subject029 | SAMD00156388 |
| Subject030 | SAMD00156389 |
| Subject031 | SAMD00156390 |
| Subject032 | SAMD00156391 |
| Subject033 | SAMD00156392 |
| Subject034 | SAMD00156393 |
| Subject035 | SAMD00156394 |
| Subject036 | SAMD00156395 |
| Subject037 | SAMD00156396 |
| Subject038 | SAMD00156397 |
| Subject039 | SAMD00156398 |
| Subject040 | SAMD00156399 |
| Subject041 | SAMD00156400 |
| Subject042 | SAMD00156401 |
| Subject043 | SAMD00156402 |
| Subject044 | SAMD00156403 |
| Subject045 | SAMD00156404 |
| Subject046 | SAMD00156405 |
| Subject047 | SAMD00156406 |
| Subject048 | SAMD00156407 |
| Subject049 | SAMD00156408 |
| Subject050 | SAMD00156409 |
| Subject051 | SAMD00156410 |
| Subject052 | SAMD00156411 |
| Subject053 | SAMD00156412 |
| Subject054 | SAMD00156413 |
| Subject055 | SAMD00156414 |
| Subject056 | SAMD00156415 |
| Subject057 | SAMD00156416 |
| Subject058 | SAMD00156417 |
| Subject059 | SAMD00156418 |
| Subject060 | SAMD00156419 |
| Subject061 | SAMD00156420 |
| Subject062 | SAMD00156421 |
| Subject063 | SAMD00156422 |
| Subject064 | SAMD00156423 |
| Subject065 | SAMD00156424 |
| Subject066 | SAMD00156425 |
| Subject067 | SAMD00156426 |
| Subject068 | SAMD00156427 |
| Subject069 | SAMD00156428 |
| Subject070 | SAMD00156429 |
| Subject071 | SAMD00156430 |
| Subject072 | SAMD00156431 |
| Subject073 | SAMD00156432 |
| Subject074 | SAMD00156433 |
| Subject075 | SAMD00156434 |
| Subject076 | SAMD00156435 |
| Subject077 | SAMD00156436 |
| Subject078 | SAMD00156437 |
| Subject079 | SAMD00156438 |
| Subject080 | SAMD00156439 |
| Subject081 | SAMD00156440 |
