# Supplementary material for: Metagenomic analyses of the gut microbiota associated with colorectal adenoma
Source: PLoS One. 2019 Feb 22;14(2):e0212406. doi: 10.1371/journal.pone.0212406 (PMC6386391; doi:10.1371/journal.pone.0212406)
Supplement: S2 Table — (DOCX) [file pone.0212406.s004.docx]

Supplementary Tale S2. Information of samples for ITS region of Fusobacterium spp. sequencing

| Subject  (name of deposit data) | BioSample No. |
| --- | --- |
| F_Subject001 | SAMD00156279 |
| F_Subject002 | SAMD00156280 |
| F_Subject003 | SAMD00156281 |
| F_Subject004 | SAMD00156282 |
| F_Subject005 | SAMD00156283 |
| F_Subject006 | SAMD00156284 |
| F_Subject007 | SAMD00156285 |
| F_Subject008 | SAMD00156286 |
| F_Subject009 | SAMD00156287 |
| F_Subject010 | SAMD00156288 |
| F_Subject011 | SAMD00156289 |
| F_Subject012 | SAMD00156290 |
| F_Subject013 | SAMD00156291 |
| F_Subject014 | SAMD00156292 |
| F_Subject015 | SAMD00156293 |
| F_Subject016 | SAMD00156294 |
| F_Subject017 | SAMD00156295 |
| F_Subject018 | SAMD00156296 |
| F_Subject019 | SAMD00156297 |
| F_Subject020 | SAMD00156298 |
| F_Subject021 | SAMD00156299 |
| F_Subject022 | SAMD00156300 |
| F_Subject023 | SAMD00156301 |
| F_Subject024 | SAMD00156302 |
| F_Subject025 | SAMD00156303 |
| F_Subject026 | SAMD00156304 |
| F_Subject027 | SAMD00156305 |
| F_Subject028 | SAMD00156306 |
| F_Subject029 | SAMD00156307 |
| F_Subject030 | SAMD00156308 |
| F_Subject031 | SAMD00156309 |
| F_Subject032 | SAMD00156310 |
| F_Subject033 | SAMD00156311 |
| F_Subject034 | SAMD00156312 |
| F_Subject035 | SAMD00156313 |
| F_Subject036 | SAMD00156314 |
| F_Subject037 | SAMD00156315 |
| F_Subject038 | SAMD00156316 |
| F_Subject039 | SAMD00156317 |
| F_Subject040 | SAMD00156318 |
| F_Subject041 | SAMD00156319 |
| F_Subject042 | SAMD00156320 |
| F_Subject043 | SAMD00156321 |
| F_Subject044 | SAMD00156322 |
| F_Subject045 | SAMD00156323 |
| F_Subject046 | SAMD00156324 |
| F_Subject047 | SAMD00156325 |
| F_Subject048 | SAMD00156326 |
| F_Subject049 | SAMD00156327 |
| F_Subject050 | SAMD00156328 |
| F_Subject051 | SAMD00156329 |
| F_Subject052 | SAMD00156330 |
| F_Subject053 | SAMD00156331 |
| F_Subject054 | SAMD00156332 |
| F_Subject055 | SAMD00156333 |
| F_Subject056 | SAMD00156334 |
| F_Subject057 | SAMD00156335 |
| F_Subject058 | SAMD00156336 |
| F_Subject059 | SAMD00156337 |
| F_Subject060 | SAMD00156338 |
| F_Subject061 | SAMD00156339 |
| F_Subject062 | SAMD00156340 |
| F_Subject063 | SAMD00156341 |
| F_Subject064 | SAMD00156342 |
| F_Subject065 | SAMD00156343 |
| F_Subject066 | SAMD00156344 |
| F_Subject067 | SAMD00156345 |
| F_Subject068 | SAMD00156346 |
| F_Subject069 | SAMD00156347 |
| F_Subject070 | SAMD00156348 |
| F_Subject071 | SAMD00156349 |
| F_Subject072 | SAMD00156350 |
| F_Subject073 | SAMD00156351 |
| F_Subject074 | SAMD00156352 |
| F_Subject075 | SAMD00156353 |
| F_Subject076 | SAMD00156354 |
| F_Subject077 | SAMD00156355 |
| F_Subject078 | SAMD00156356 |
| F_Subject079 | SAMD00156357 |
| F_Subject080 | SAMD00156358 |
| F_Subject081 | SAMD00156359 |
